# The preventive efficacy of vitamin B supplements on the cognitive decline of elderly adults: a systematic review and meta-analysis

**DOI:** 10.1186/s12877-021-02253-3

**Published:** 2021-06-16

**Authors:** Shufeng Li, Yuchen Guo, Jie Men, Hanlin Fu, Ting Xu

**Affiliations:** 1 Department of Health Statistics, Fenyang College of Shanxi Medical University, 032200 Fenyang, Shanxi China; 2 Fuwai Hospital, Chinese Academy of Medical Sciences, Xicheng District 100032, Beijing, China; 3 Department of Basic Medicine, Fenyang College of Shanxi Medical University, 032200 Fenyang, Shanxi China; 4 Department of Epidemiology and Health Statistics, XiangYa School of Public Health, Central South University, 410008 Changsha, Hunan China; 5 Department of Physiology, Fenyang College of Shanxi Medical University, 032200 Fenyang, Shanxi China

**Keywords:** Preventive efficacy, Vitamin B, Cognitive function, Randomized controlled trials, Elderly adults, Meta-analysis

## Abstract

**Background:**

The irreversibility of cognitive impairment of Alzheimer’s disease (AD) prompts that preventing or delaying the onset of AD should be a public health priority. Vitamin B supplements can lower the serum homocysteine (Hcy) level, but whether it can prevent cognitive decline or not remains unclear. We aimed to evaluate the preventive efficacy of vitamin B supplements on the cognitive decline of elderly adults.

**Methods:**

We searched PubMed, Embase, The Cochrane Central Register of Controlled Trials (CENTRAL), Web of Science, Scopus, Science Direct, PsycINFO from inception to December 1, 2019, and then updated the retrieved results on June 1, 2020. The randomized controlled trials (RCTs) which evaluated the efficacy of vitamin B in mild cognitive impairment (MCI) patients or elderly adults without cognitive impairment were selected. Standardized mean difference (SMD) or mean difference (MD) as well as their 95 % confidence interval (CI) were calculated by performing random effects models or fixed effects models.

**Results:**

A total of 21 RCTs involving 7571 participants were included for meta-analysis. The forest plots showed that there is significant effect in global cognitive function (15 RCTs, SMD: 0.36; 95 % CI: 0.18 to 0.54, P < 0.01) and Hcy (11 RCTs, MD: -4.59; 95 %CI: -5.51 to -3.67, P < 0.01), but there is no effect in information processing speed (10 RCTs, SMD: 0.06; 95 % CI: -0.12 to 0.25, P = 0.49), episodic memory (15 RCTs, SMD: 0.10; 95 % CI: -0.04 to 0.25, P = 0.16), executive function (11 RCTs, SMD: -0.21; 95 % CI: -0.49 to 0.06, P = 0.13). The value of effect size and heterogeneity did not vary apparently when excluding the low-quality studies, so we could believe that the results of meta-analysis were robust.

**Conclusions:**

Vitamin B supplements might delay or maintain the cognitive decline of elderly adults. We can recommend that the vitamin B supplements should be considered as a preventive medication to MCI patients or elderly adults without cognitive impairment. More well-designed RCTs with large sample sizes were required to clarify the preventive efficacy in the future.

**Supplementary Information:**

The online version contains supplementary material available at 10.1186/s12877-021-02253-3.

## Background

Alzheimer’s disease (AD) is the main cause that leads to cognitive decline in elderly adults [1]. With the population aging, the morbidity of AD rises rapidly among elderly adults. The Alzheimer’s Disease International (ADI) estimates that there are currently over 50 million people living with dementia globally and there will be about 152 million dementia patients by 2050 [2]. The health-care costs and economy burden of AD is enormous [3]. Furthermore, the total costs are closely related to the different stages (mild, moderate, severe) and rises significantly with the severity of AD [4]. Mild cognitive impairment (MCI) is clinically significant memory impairment that does not meet the criteria for AD and could be regarded as the preclinical stages of AD. MCI as the transitional stage between normal cognitive aging and AD [5, 6], of which 5–10 % will progress to AD [7]. The progression of MCI to AD is complex multifactorial degenerative process and the trajectory of this process is susceptible to some extent [8]. A study based on a mathematical model reported that delaying the onset of AD by 5 years would result in a 57 % reduction in the number of AD patients and reduce the medical costs of AD from $627 to $344 billion dollars [9]. Therefore, much attention should be focused on the modifiable risk factors and effective intervention to prevent or delay the progression of MCI to AD and preventing or delaying the onset of AD should be a public health priority.

The main pathological characteristics of AD are the amyloid plaques due to the accumulation of β-amyloid peptide (Aβ) and the neurofibrillary tangles (NFTs) that contains hyperphosphorylated microtubule-associated tau proteins [10, 11]. Homocysteine (Hcy) is a neurotoxic amino acid as a by-product of methionine transmethylation, which can cause the accumulation of Aβ and brain atrophy [12]. Vitamin B12, folate and vitamin B6 are cofactors for the methylation of Hcy and play a vital role in lowering the levels of serum Hcy [13]. Low serum status of vitamin B and high Hcy levels may cause brain atrophy through oxidative stress and lead to the cognitive decline of elderly adults [14–17]. The elimination of excess Hcy could be a potential therapeutic intervention to improve cognitive function or delay the onset of AD [18]. Folate and the metabolically related vitamin B are considered promising for preventing or delaying aged-related cognitive decline to people with high serum levels of Hcy [19–21].

There have been several systematic reviews evaluating the efficacy vitamin B for AD or MCI. A recent systematic review published in 2019 evaluated the efficacy of treatment with vitamin B in slowing cognitive decline among elderly adults with and without cognitive impairment by the outcome measure of Mini-Mental State Examination scores (MMSE) [22]. However, MMSE as the only outcome measure utilized in this review, it cannot evaluate the therapeutic efficacy comprehensively. There are other several existing resembling systematic reviews which evaluated vitamin B12, vitamin B6, or folic acid alone or in combination on cognitive function in adults with either normal or impaired cognitive function. However, they focused on therapeutic efficacy rather than preventive efficacy [23–27]. Furthermore, several most recent RCTs had not been systematically reviewed [28–30].

Up to now, there is as yet no effective medication to improve the cognitive function by alter the course of AD [31]. In view of the irreversibility of cognitive impairment, we should pay more attention to MCI patient or elderly adults without cognitive impairment rather than AD patients. Meanwhile, several randomized controlled trials (RCTs) concerning evaluating the preventive efficacy of vitamin B supplements on the cognitive decline of elderly adults were screened, but the conclusions are inconsistent. Therefore, it is necessary to conduct a systematic review and meta-analysis to verify the preventive efficacy. We expected that our research results will assist in guiding clinicians and health educator to optimize the prescription patterns for the MCI patients and elderly adults without cognitive impairment.

## Methods

This systematic review and meta-analysis was conducted according to Preferred Reporting Items for Systematic Reviews and Meta-Analyses (PRISMA) guidelines [32] and Cochrane Handbook for Systematic reviews for Interventions [33]. The PRISMA Checklist was presented in Additional file 1.

### Search strategy

We intended to include all RCTs that compared the preventive efficacy of vitamin B supplements with placebo in MCI patients or elderly adults without cognitive impairment. We searched PubMed, Embase, The Cochrane Central Register of Controlled Trials (CENTRAL), Web of Science, Scopus, Science Direct, PsycINFO from inception to December 1, 2019 and then updated the retrieved results on June 1, 2020. The main retrieval approaches we used were standardized subject terms (medical subject headings (Mesh) in PubMed, Emtree terms in Embase), free text words, Boolean logic, truncation operator and search fields retrieval. The reference lists of previous systematic reviews and relevant articles were cross-checked to identify any potentially relevant studies that met our inclusion criteria as well. The detailed information of search strategy was listed in the Additional file 2.

### Inclusion and exclusion criteria

Studies were included if they met all the following inclusion criteria: (1) a placebo-controlled randomized controlled trial; (2) MCI patients or elderly adults without cognitive impairment; (3) the age of participants > 50 years; (4) with or without comorbidity of chronic disease, such as transient ischemic, diabetes, hyperhomocysteinemia, unstable angina, hypertension and so on; (5) intervention with vitamin B12, vitamin B6, or folic acid alone or in combination in any form, frequency, dosage; (6) used one or more outcome measures of cognitive function; (7) articles published in English.

Studies were excluded if they met one of the following exclusion criteria: 1**)** participants were diagnosed as Alzheimer’s disease or other forms of dementia; (2) patients with cognitive decline due to head trauma or brain tumor; (3) letters, comments, case reports, editorials, animal studies.

### Selection of studies and data extraction

Merged the records which retrieved from the different sources and removed the duplicate records using the software of EndNote X9. Two investigators (Li and Xu) independently screened the title and abstract of the records mentioned above and then removed obviously irrelevant records. Read the full text of the potentially relevant records to determine eligibility for inclusion. If multiple resembling publications were derived from the same trial, kept the latest one. When it comes to disagreements, consulted with each other or turned to a third investigator (Fu) for judgement.

The two investigators extracted the following data independently and then listed into the Table 1: first author, year of publication, country, participants, the baseline levels of serum Hcy, characteristics of participants (intention-to-treat (ITT) population, mean age, the sample size and proportion of male), intervention (different types and combinations of vitamin B, frequency, dosage), treatment duration. When the important information needed for meta-analysis was unavailable, we sent emails to the responsible authors for help. We extracted the mean and SD of continuous variables from the graphs by utilizing the software of GetData Graph Digitizer 2.26 (http://www.getdata-graph-digitizer.com) when the valid data was presented only in graphs.

**Table 1:** Summary of characteristics of RCTs included in the systematic review

First Author (Year) Country	Participants	Baseline levels of serum Hcy ^a^ (µmol/L)	Characteristics of Participants			Intervention	Treatment Duration
			N(IG/CG) ITT	Mean Age ^a^ (years) (IG/CG)	Male (n/%) (IG/CG)		
Fei Ma (2019) China [28]	120 MCI patients aged ≥ 65 years with no terminal illness or mental disorders, recruited from communities	20.6 ± 2.2	60/60	69.2 ± 2.5/ 68.5 ± 3.9	21(35.0)/ 22(36.7)	folic acid 800 µg and vitamin B12 25 µg/day	6 months
Kwok Timothy (2019) China [29]	279 MCI outpatients aged ≥ 65 years	13.8 ± 3.2	138/141	76.9 ± 5.4/ 78.0 ± 5.3	87(63.1)/ 79(56.1)	folic acid 400 µg and vitamin B12 500 µg/day	24 months
Fei Ma (2017) China [30]	180 MCI patients aged ≥ 65 years with no terminal illness or mental disorders, recruited from communities	12.9 ± 4.4	90/90	74.8 ± 2.8/ 74.6 ± 3.2	39(43.3)/ 38(42.2)	folic acid 400 µg/day	24 months
Kwok Timothy (2017) China [34]	271 diabetic non-demented outpatients aged ≥ 70 years	16.8 ± 5.7	137/134	74.7 ± 4.0/ 75.8 ± 4.2	82(59.9)/ 76(56.7)	vitamin B12 1000 µg/day	27 months
Cheng D (2016) China [35]	104 elderly volunteers aged ≥ 55 years, recruited from communities or nursing homes	20.0 ± 5.6	57/47	74.3 ± 9.6/ 72.5 ± 7.0	27(47.4)/ 23(48.9)	folic acid 800 µg, vitamin B6 10 mg and vitamin B12 25 µg/day	14 weeks
Dangour AD (2015) United Kingdom [36]	201 healthy volunteers aged ≥ 75 years	16.1 ± 4.3	99/102	79.9 ± 3.5/ 80.1 ± 3.7	46(46.5)/ 23(48.9)	vitamin B12 1 mg/day	12 months
van der Zwaluw NL (2014) Netherlands [37]	856 elderly participants aged ≥ 65 years with no dementia	14.2 ± 2.5	425/431	72.6 ± 5.7/ 72.6 ± 5.8	244(57.0)/ 255(59.0)	folic acid 400 µg and vitamin B12 500 µg/day	24 months
Hankey GJ (2013) Australia [26]	2214 patients with stroke or transient ischemic attack, MMSE ≥ 24	14.3 ± 8.5	1110/1104	63.6 ± 11.8 ^b^	1491(67.3)	folic acid 2 mg, vitamin B6 25 mg and vitamin B12 500 µg/day	3.4 years
Walker JG (2012) Australia [38]	900 elderly adults aged 60 ~ 74 years, recruited from communities	9.7 ± 2.7	447/453	65.9 ± 4.3/ 66.0 ± 4.2	181(40.5)/ 177(39.1)	folic acid 400 µg and vitamin B12 100 µg/day	24 months
de Jager CA (2012) United Kingdom [39]	266 MCI patients aged ≥ 70 years	11.5 ± 4.5	133/133	76.8 ± 5.1/ 76.7 ± 4.8	40(36.4)/ 40(35.4)	folic acid 0.8 mg, vitamin B12 0.5 mg and vitamin B6 20 mg/day	24 months
Ford AH (2010) Australia [40]	299 community-dwelling elderly men aged ≥ 75 years with hypertension	13.6 ± 5.3	150/149	79.3 ± 2.8/ 78.7 ± 2.7	150(100)/ 149(100)	folic acid 2 mg, vitamin B12 0.4 mg and vitamin B6 25 mg/day	24 months
van Uffelen JG (2008) Netherlands [41]	179 MCI patients aged 70 ~ 80 years, recruited from communities	NR	90/89	75.4 ± 2.8/ 74.9 ± 3.0	44(56.4)/ 41(55.4)	folic acid 5 mg, vitamin B12 0.4 mg and vitamin B6 50 mg/day	12 months
Durga J (2007) Netherlands [42]	819 men and post-menopausal women aged 50 ~ 70 years	12.9 ± 2.4	406/413	60 ± 5/ 60 ± 6	294(72)/ 292(70)	folic acid 0.8 mg/day	3 years
McMahon JA (2006) New Zealand [43]	276 healthy participants aged ≥ 65 years	16.6 ± 4.9	138/138	73.6 ± 5.8/ 73.4 ± 5.7	80(63.0)/ 61(48.4)	folic acid 1 mg, vitamin B12 0.5 mg and vitamin B6 10 mg/day	2 years
Eussen SJ (2006) Netherlands [44]	131 elderly people aged ≥ 70 years with mild vitamin B12 deficiency	15.2 ± 5.1	66/65	83 ± 6/ 82 ± 5	49(74.2)/ 51(78.5)	folic acid 0.4 mg, vitamin B12 1 mg/day	6 months
Stott DJ (2005) United Kingdom [45]	47 elderly people aged ≥ 65 years with ischemic vascular disease	16.7 ± 6.5	23/24	72.6 ± 6.4/ 72.8 ± 5.4	12(52.2)/ 14(58.3)	folic acid 2.5 mg, vitamin B12 0.5 mg and vitamin B6 25 mg/day	3 months
Lewerin C (2005) Sweden [46]	195 community-dwelling participants	17.2 ± 5.2	126/69	75.7 ± 4.7/ 75.6 ± 4.0	48(38.1)/ 30(43.5)	folic acid 0.8 mg, vitamin B12 0.5 mg and vitamin B6 3 mg/day	4 months
Hvas AM (2004) Denmark [47]	140 elderly people with plasma methylmalonic acid 0.40 ~ 2.00µmol/L	13.2 ^c^	70/70	75/74 ^c^	20(28.6)/ 22(31.4)	vitamin B12 1 mg/week	1 month
Garcia A (2004) Canada [48]	24 community-dwelling participants aged ≥ 65 years, with an increased plasma methylmalonic acid and MMSE ≥ 24	12.2 ± 4.0	12/12	76/76 ^c^	5(22.7) ^b^	vitamin B12 1 mg/month	6 months
Janet Bryan (2002) Australis [49]	40 healthy older women aged 65 ~ 92 years, who did not smoke, were not pregnant or lactating	NR	19/21	74.1 ± 5.75 ^b^	0(0)/ 0(0)	folic acid 750 µg/day	5 weeks
Fioravanti M (1998) Italy [50]	30 elderly patients aged 70 ~ 90 years, with memory complaints, MMSE score 16 ~ 24 and serum folate < 3 ng/ml, recruited from homes or communities	NR	16/14	80.3 ± 5.8/ 80.2 ± 5.5	4(25)/ 1(7.1)	folic acid 15 mg/day	2 months

*Abbreviations*: *IG* intervention group, *CG* control group, *ITT* Intention-To-Treat, *NR* not reported

^a^ Mean ± SD; ^b^ the combination of intervention group and control group; ^c^ the SD is unavailable

### Quality assessment

Two investigators (Li and Xu) independently assessed the risk of bias of each included study according to the Cochrane risk-of-bias tool [51]. This tool assesses the following risk of bias domains: random sequence generation (selection bias); allocation concealment (selection bias); blinding of participants and personnel (performance bias); blinding of outcome assessment (detection bias); incomplete outcome data (attrition bias); selective reporting (reporting bias); other bias. The judgement to the risk of bias of each domain can be categorized into low risk, high risk, or unclear bias. Any disagreements were resolved by consensus or turned to the third investigator (Fu) for judgement. The low-quality assessment of studies included was not an exclusion criterion.

### Definitions of outcome measures

The studies involved substantial and different cognitive function scales to assess the preventive efficacy of vitamin B supplements, so the majority of cognitive function scales were involved in only a few of studies. If we set every cognitive function scale mentioned above as an outcome measure for the meta-analysis, it would induce only a few of studies were included in every outcome measure for meta-analysis. To avoid this problem and get a more convincing and robust result, we classified each cognitive function scale into one of four categories: (1) global cognitive function; (2) information processing speed; (3) episodic memory; (4) executive function. Additional file 3 lists the cognitive function scales involved in studies and shows their cognitive domain categories.

### Statistical Analysis

 We performed the meta-analysis by using the version 5.3 of Review Manager (The Nordic Cochrane Centre, Copenhagen). The forest plot for continuous data based on mean change from baseline rather than post-intervention values. If SD of mean change (SD_*change*_) is not presented, we assumed a particular correlation coefficient (Corr) which describes the relationship between the SD of baseline (SD_*baseline*_) and SD of final (SD_*final*_). This *Corr* can be obtained from the study [42] which lists all of the SD_*baseline*_, SD_*baseline*_ and SD_*change*_ in its paper. we attempted to calculate SD_*change*_ by the following formula: ${\text{SD}}_{\text{change}}\text{=}\sqrt{{\text{SD}}_{\text{baseline}}^{\text{2}}\text{+}{\text{SD}}_{\text{final}}^{\text{2}}\text{-(2*}\text{Corr}\text{*}{\text{SD}}_{\text{baseline}}\text{*}{\text{SD}}_{\text{final}}\text{)}}$. Nonetheless, if the Corr of some outcome measures cannot be obtained from any of the studies included, we assumed the Corr of 0.5 between the SD_*baseline*_ and SD_*final*_.

The standardized mean difference (SMD) of change scores as well as its 95 % confidence interval (CI) was used when it comes to an outcome measure which contained several different cognitive function scales; The mean difference (MD) of change scores as well as its 95 % CI was used when it comes to an outcome measure which contained only an identical measurement indicators (for instance Hcy).

Statistical heterogeneity of intervention efficacy was assessed using the *P* value from the chi-squared test combined with the I-square statistic. If the *P* > 0.10 and I-square statistic *<* 50 %, we considered the heterogeneity to be moderate and used a fixed effects model which ignored heterogeneity; If the *P <* 0.10 or I-square statistic > 50 %, we considered the heterogeneity to be substantial and used a random effects model to obtain a relatively conservative intervention evaluation effect.

In this case, the sources of heterogeneity can be explored by performing subgroup analysis. The subgroup analysis can base on the characteristic as follows: (1) treatment duration: short (≤ 12 months) contrast long (> 12months); (2) participants: MCI patients contrast elderly adults without cognitive impairment. We assessed the robustness of results by excluding the low-quality studies and then compared the results to previous pooled estimate. We reported the results only if the pooled estimates varied significantly.

In most of the cognitive function scales, higher cognitive scores indicate better cognitive function. But there are exceptions, in cases where they were reversed, we multiplied the cognitive scores by − 1. The levels of serum Hcy in included studies used the conventional unit (ng/ml) or Systeme International (SI) unit (µmol/L). we multiplied the conventional unit by the formula weight of Hcy and then got the SI unit.

## Results

### Overall search findings

**Fig. 1:**
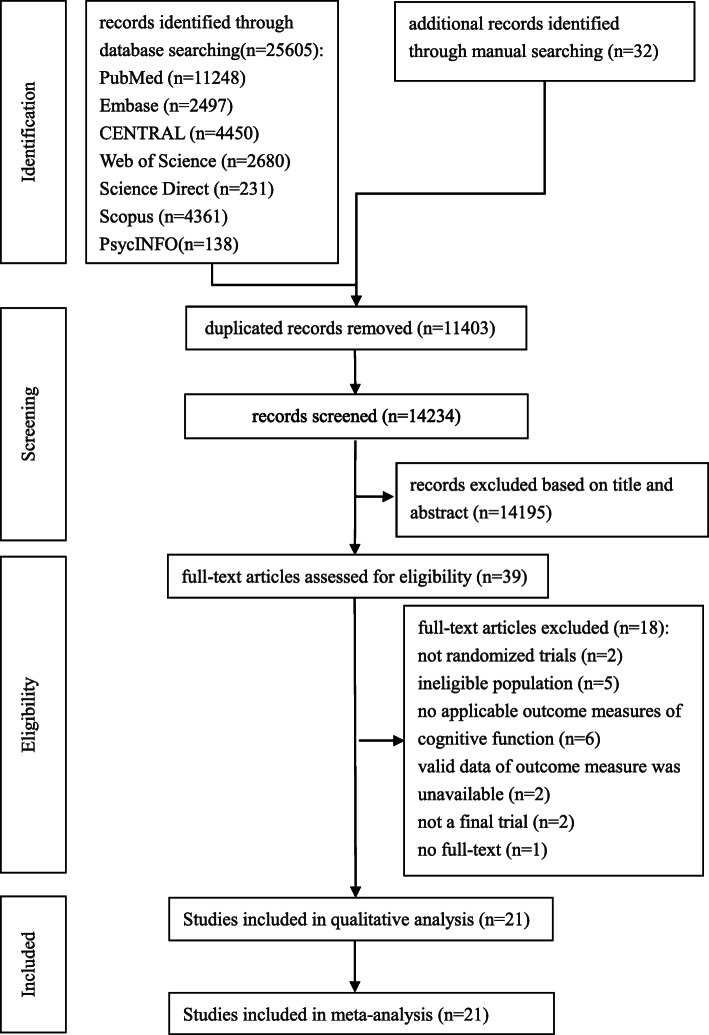
Flow chart for the identification of studies

A total of 25,637 records were retrieved, 25,605 from the seven electronic databases and 32 from manual searching. After removed the duplicate records and obviously irrelevant records, 39 records remained. Read the full text then 18 records were excluded. The 18 excluded studies as well as the reasons for exclusion were listed in Additional file 4. A total of 21 eligible RCTs were included for meta-analysis by reading the full text of the potentially relevant records. The flow chart for the identification of studies was presented in Fig. 1.

### Characteristics of included studies

These studies were originated from nine countries and were published between 1998 and 2019. The sample sizes of included studies ranged from 24 to 2214. A total of 7571 participants were included in systematic review, of which 3812 were in the intervention group and 3759 were in the control group. They were recruited from homes, communities or hospitals. The participants of six studies [28–30, 39, 41, 50] were MCI patients and the other fifteen studies [26, 34–38, 40, 42–49] were elderly adults without cognitive impairment. Some of these participants were accompanied with one or more chronic diseases, such as mental disorders, stroke, diabetes mellitus, transient ischemic attack or hypertension. The baseline levels of serum Hcy of participants included were high (ranged from 9.7 ~ 20.6 µmol/L). The mean age of participants ranged from 60.0 to 83 in the intervention group and 60.0 to 82 in the control group. The treatment duration ranged from 1 month to 3.4 years, eleven studies [28, 35, 36, 41, 44–50] ≤ 12 months and ten studies [26, 29, 30, 34, 37–40, 42, 43] > 12 months. The detail characteristics of 21 included studies were presented in Table 1.

### Quality assessment of included studies (risk of bias)

**Fig. 2:**
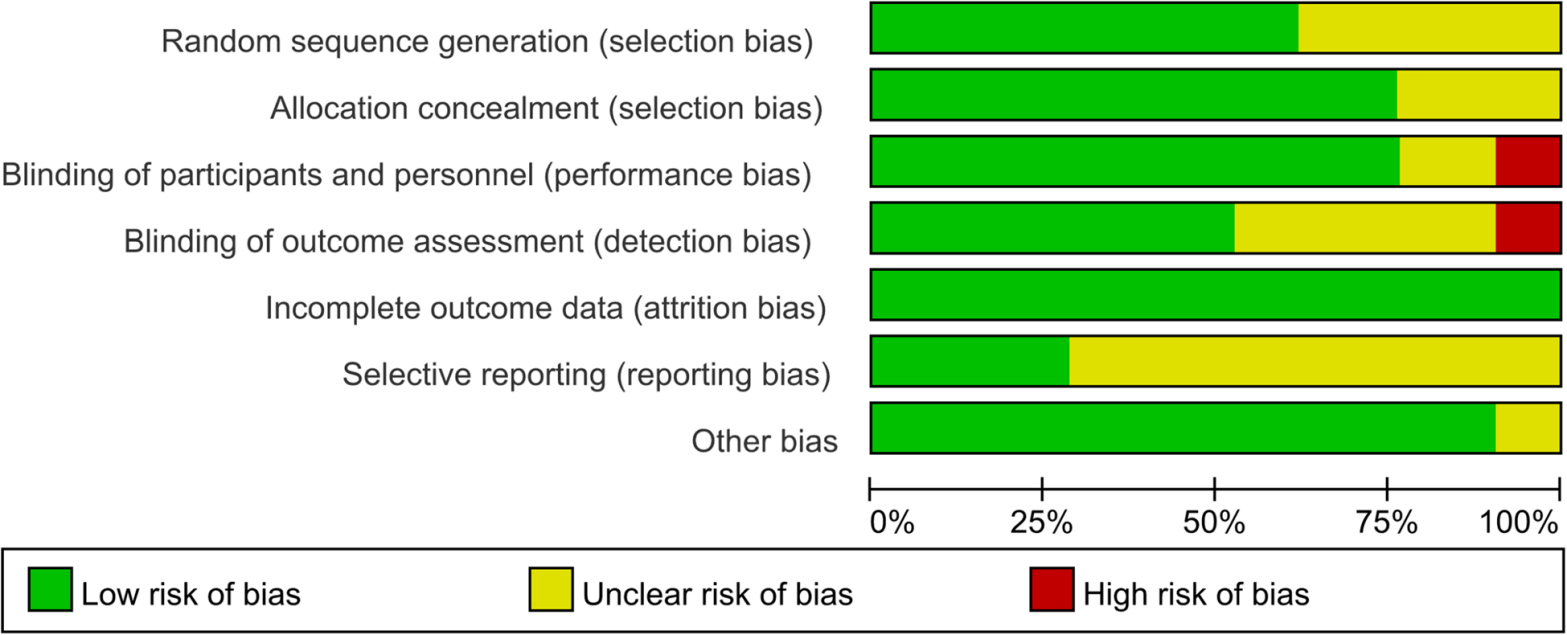
Risk of bias graph: the judgements about each risk of bias item presented as percentages

The risk of bias of included studies were summarized in Fig. 2. Overall, the selection bias of the 21 studies included was reasonable: 13/21 of the studies included were assessed to have a low risk of bias for random sequence generation; 14/21 of the studies included were assessed to have a low risk of bias for allocation concealment. The study conducted by Fei Ma which published in 2019[28] had a high risk of bias for blinding of assessors, participants and providers. Similarly, another study conducted by Fei Ma which published in 2017[30] had a high risk of bias for blinding of assessors. All the studies included had a low risk of bias for incomplete outcome data. The six studies included [26, 37, 39–42] were assessed to have a low risk of bias for selective reporting, and the protocols of the rest studies included were unavailable. The quality assessment results of risk of bias were presented in Additional file 5.

### Outcome evaluation

Four outcome measures of cognitive function (global cognitive function, information processing speed, episodic memory, executive function) and the levels of serum Hcy were used to evaluate the preventive efficacy of vitamin B supplements in MCI patients or elderly adults without cognitive impairment. We presented the detailed results of meta-analysis as follows.

#### Global cognitive function

**Fig. 3:**
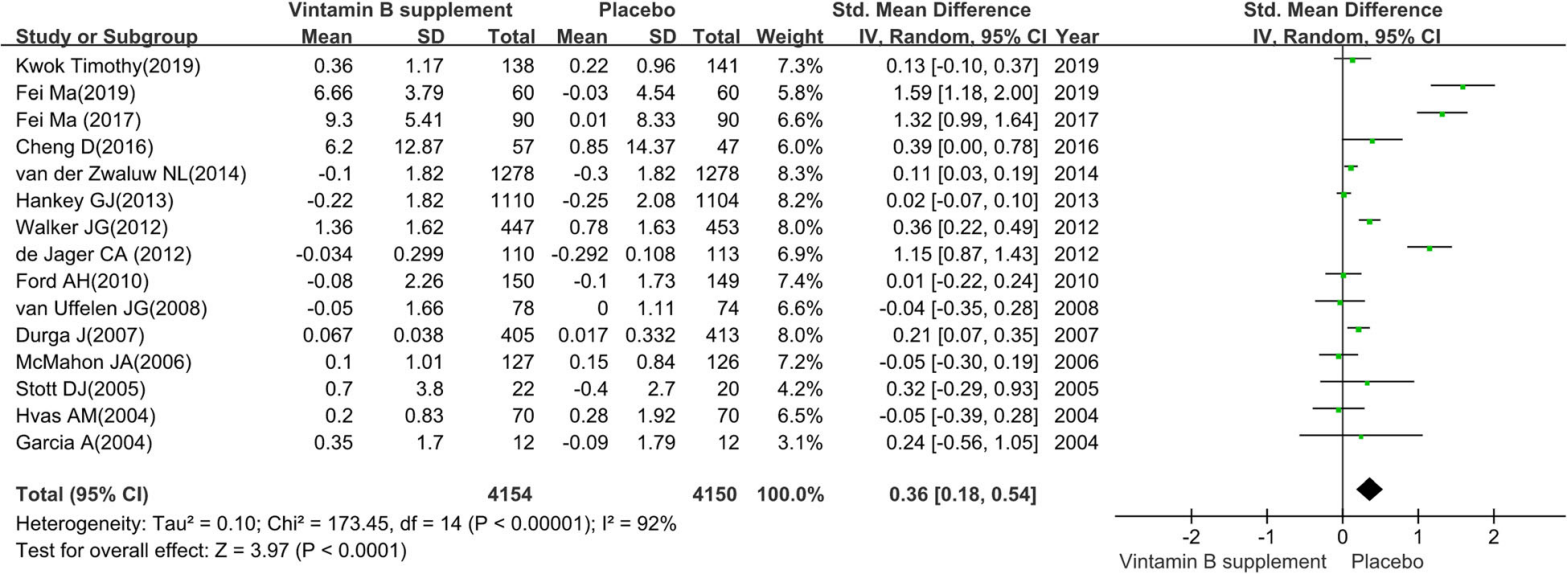
The preventive efficacy of vitamin B supplements versus placebo on global cognitive function

We pooled the data extracted from 15 RCTs [26, 28–30, 35, 37–43, 45, 47, 48] and found a substantial heterogeneity (heterogeneity test: Chi-square = 173.45, df = 14, P < 0.01, I-square = 92 %). The intervention group achieved significant preventive efficacy on global cognitive function (SMD: 0.36; 95 % CI: 0.18 to 0.54, P < 0.01) by using a random-effects model (see Fig. 3).

The sensitivity analysis revealed the pooled SMD as well as its 95 %CI of global cognitive function was robust. The obvious overlap of the 95 %CI of short treatment duration (≤ 12 months) contrast long treatment duration (> 12months) and the test for subgroup differences (P = 0.67) indicated that there was no interaction between the pooled SMD and treatment duration. On the contrary, the negligible overlap of the 95 %CI of MCI patients contrast elderly adults without cognitive impairment and the test for subgroup differences (P = 0.03) indicated that there was interaction between the pooled SMD and participants. The detailed results of subgroup analysis were presented in Table 2.

**Table 2:** The results of subgroup analysis

Outcome measures	Subgroup		The Num of studies	Pooled estimate [SMD/MD (95 %CI)]	P value	*I*^2^(%)	Test for subgroup differences
global cognitive function	Treatment duration	≤ 12 months	7	0.35(-0.15, 0.84)	P = 0.17	87	P = 0.67
		> 12 months	8	0.23(0.07, 0.40)	P = 0.006	91	
	participants	MCI patients	5	0.82(0.20, 1.45)	P = 0.01	95	P = 0.03*
		EAWCI	10	0.13(0.03, 0.23)	P = 0.01	66	
information processing speed	Treatment duration	≤ 12 months	5	-0.19(-0.38, 0.01)	P = 0.06	39	P = 0.007*
		> 12 months	4	0.16(0.01, 0.31)	P = 0.04	64	
	participants	MCI patients	1	0.04(-0.28, 0.36)	P = 0.79	NA	P = 0.89
		EAWCI	9	0.07(-0.13, 0.27)	P = 0.50	81	
episodic memory	Treatment duration	≤ 12 months	7	-0.09(-0.27, 0.09)	P = 0.34	40	P = 0.09
		> 12 months	6	0.08(0.02, 0.13)	P = 0.01	0	
	participants	MCI patients	4	0.42(-0.24, 1.08)	P = 0.21	94	P = 0.26
		EAWCI	11	0.04(-0.04, 0.12)	P = 0.34	28	
executive function	Treatment duration	≤ 12 months	4	0.01(-0.14, 0.17)	P = 0.87	0	P = 0.21
		> 12 months	5	-0.29(-0.75, 0.16)	P = 0.20	96	
	participants	MCI patients	3	-0.33(-0.81, 0.15)	P = 0.18	89	P = 0.59
		EAWCI	8	-0.17(-0.51, 0.17)	P = 0.34	94	
Hcy	Treatment duration	≤ 12 months	7	-4.77(-6.28, -3.27)	P < 0.01	78	P = 0.68
		> 12 months	5	-4.41(-5.27, -3.54)	P < 0.01	82	
	participants	MCI patients	3	-5.45(-6.51, -4.39)	P < 0.01	81	P = 0.05
		EAWCI	8	-4.13(-4.93, -3.32)	P < 0.01	65	

*Abbreviations*: *SMD* standardized mean difference, *MD* mean difference, *CI* confidence interval,*MCI* mild cognitive impairment, *EAWCI* elderly adults without cognitive impairment, *Hcy* Homocysteine, *NA* not applicable

^*^P < 0.05

#### Information processing speed

**Fig. 4:**
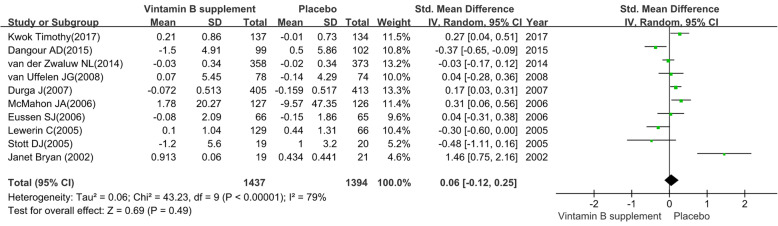
The preventive efficacy of vitamin B supplements versus placebo on information processing speed

We pooled the data extracted from 10 RCTs [34, 36, 37, 41–46, 49] and found a substantial heterogeneity (heterogeneity test: Chi-square = 43.23, df = 9, P < 0.01, I-square = 79 %). The intervention group achieved no preventive efficacy on information processing speed (SMD: 0.06; 95 % CI: -0.12 to 0.25, P = 0.49) by using a random-effects model (see Fig. 4).

The sensitivity analysis revealed the pooled SMD with its 95 %CI of information processing speed was robust. The non-overlap of the 95 %CI of short treatment duration (≤ 12 months) contrast long treatment duration (> 12months) and the test for subgroup differences (P = 0.007) indicated that there was interaction between the pooled SMD and treatment duration. On the contrary, the obvious overlap of the 95 %CI of MCI patients contrast elderly adults without cognitive impairment and the test for subgroup differences (P = 0.89) indicated that there was no interaction between the pooled SMD and participants. The detailed results of subgroup analysis were presented in Table 2.

#### Episodic memory

**Fig. 5:**
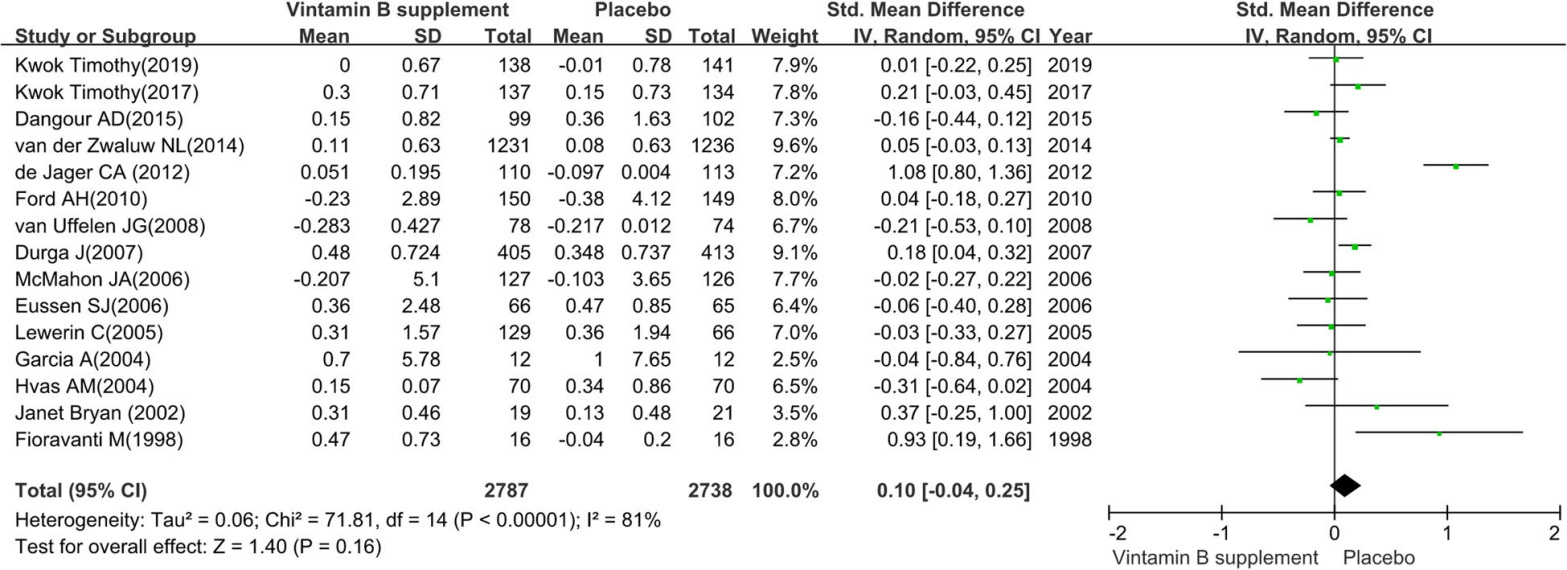
The preventive efficacy of vitamin B supplements versus placebo on episodic memory

We pooled the data extracted from 15 RCTs [29, 34, 36, 37, 39–44, 46–50] and found a substantial heterogeneity (heterogeneity test: Chi-square = 71.81, df = 14, P < 0.01, I-square = 81 %). The intervention group achieved no preventive efficacy on episodic memory (SMD: 0.10; 95 % CI: -0.04 to 0.25, P = 0.16) by using a random-effects model (see Fig. 5).

The sensitivity analysis revealed the pooled SMD with its 95 %CI of episodic memory was robust. The obvious overlap of the 95 %CI of short treatment duration (≤ 12 months) contrast long treatment duration (> 12months) and the test for subgroup differences (P = 0.09) indicated that there was no interaction between the pooled SMD and treatment duration. Similarly, the obvious overlap of the 95 %CI of MCI patients contrast elderly adults without cognitive impairment and the test for subgroup differences (P = 0.26) indicated that there was no interaction between the pooled SMD and participants. The detailed results of subgroup analysis were presented in Table 2.

#### Executive function

**Fig. 6:**
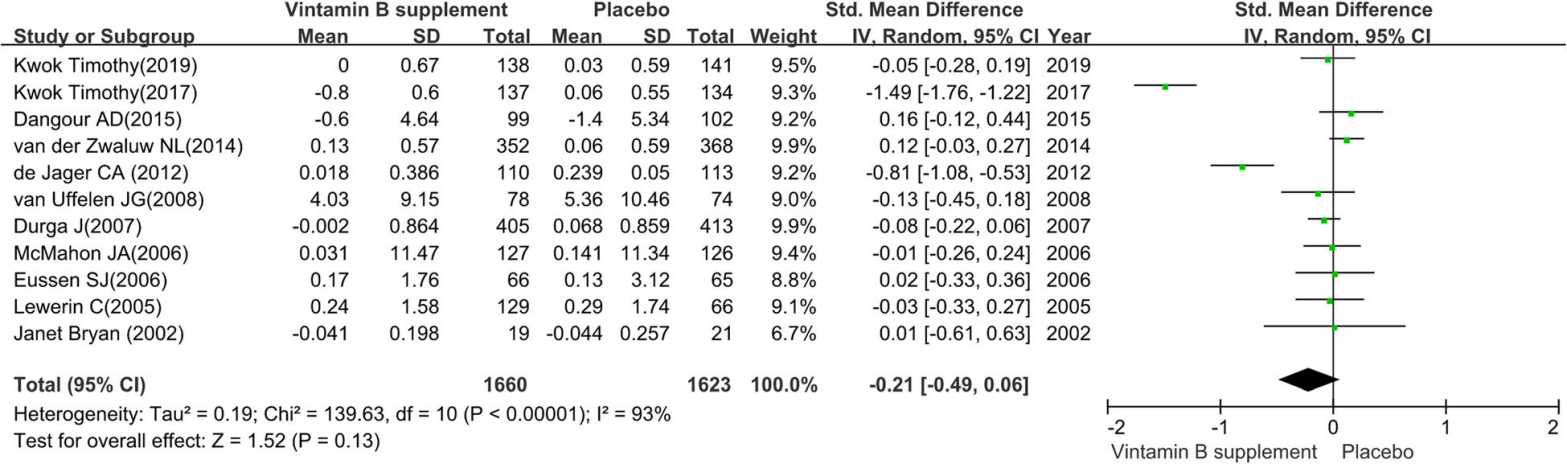
The preventive efficacy of vitamin B supplements versus placebo on executive function

We pooled the data extracted from 11 RCTs [29, 34, 36, 37, 39, 41–44, 46, 49] and found a substantial heterogeneity (heterogeneity test: Chi-square = 139.63, df = 10, P < 0.01, I-square = 93 %). The intervention group achieved no preventive efficacy on executive function (SMD: -0.21; 95 % CI: -0.49 to 0.06, P = 0.13) by using a random-effects model (see Fig. 6).

The sensitivity analysis revealed the pooled SMD with its 95 %CI of executive function was robust. The obvious overlap of the 95 %CI of short treatment duration (≤ 12 months) contrast long treatment duration (> 12months) and the test for subgroup differences (P = 0.21) indicated that there was no interaction between the pooled SMD and treatment duration. Similarly, the obvious overlap of the 95 %CI of MCI patients contrast elderly adults without cognitive impairment and the test for subgroup differences (P = 0.59) indicated that there was no interaction between the pooled SMD and participants. The detailed results of subgroup analysis were presented in Table 2.

#### Hcy

**Fig. 7:**
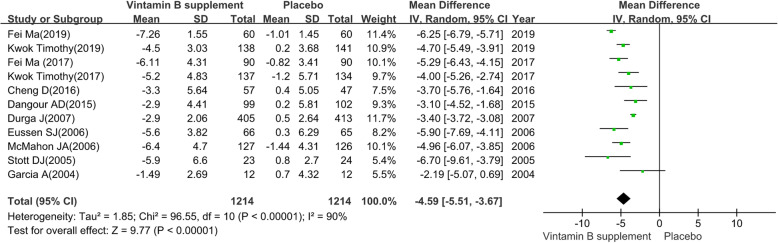
The preventive efficacy of vitamin B supplements versus placebo on Hcy

We pooled the data extracted from 11 RCTs [28–30, 34–36, 42–45, 48] and found a substantial heterogeneity (heterogeneity test: Chi-square = 96.55, df = 10, P < 0.01, I-square = 90 %). The intervention group achieved significant preventive efficacy on Hcy (MD: -4.59; 95 % CI: -5.51 to -3.67, P < 0.01) by using a random-effects model (see Fig. 7).

The sensitivity analysis revealed the pooled MD with 95 %CI of global cognitive function were robust. The obvious overlap of the 95 %CI of short treatment duration (≤ 12 months) contrast long treatment duration (> 12months) and the test for subgroup differences (P = 0.680) indicated that there was no interaction between the pooled SMD and treatment duration. On the contrary, the non-overlap of the 95 %CI of MCI patients contrast elderly adults without cognitive impairment and the test for subgroup differences (P = 0.05) indicated that there was interaction between the pooled SMD and participants. The detailed results of subgroup analysis were presented in Table 2.

## Discussion

### Summary of results

This systematic review explored the preventive efficacy of vitamin B supplements on the cognitive decline of MCI patients or elderly adults without cognitive impairment by synthesizing 21 eligible RCTs. We found that vitamin B supplements can significantly lower the levels of serum Hcy and prevent the decline of global cognitive function. The substantial reduction of the levels of serum Hcy verified that vitamins B are cofactors for the methylation of Hcy and play a vital role in lowering the levels of serum Hcy. Besides, the high levels of serum Hcy of participants may also be a crucial factor. In view of this, we can draw the conclusions that vitamin B supplements can prevent or delay aged-related cognitive decline by lowering the levels of Hcy. As for the other three outcome measures (information processing speed, episodic memory, executive function), the vitamin B supplements was invalid. The difference in conclusions may be related to the assumption that the vitamin B supplements have efficacy on global cognitive function rather than the other three outcome measures. Besides, the assumption that the outcome measure of global cognitive function was more sensitive than the other three outcome measures to detect the change of cognitive function may also be related to the difference.

The heterogeneity of every outcome measure was substantial and was caused by various reasons. First, both MCI patients and elderly adults without cognitive impairment were included in our meta-analysis. Because of age-related of degeneration, elderly adults were at high risk of chronic disease, such as mental disorders, stroke, diabetes mellitus, transient ischemic attack or hypertension. Different chronic diseases may influence the metabolism of vitamin B or the assessment of cognitive function; second, the baseline levels of serum Hcy of participants varied significantly. The therapeutic efficacy of vitamin B supplements in participants was significantly associated with the baseline levels of serum Hcy [52]; third, the treatment duration of studies included ranged from 1 month to 3.4 years. It is generally believed that longer treatment duration is more likely to achieve better therapeutic efficacy; last, the cognitive function scales utilized in studies were diversified. The 21 included studies involved 53 different cognitive function scales in total. One outcome measure was assessed by several cognitive function scales in most cases.

We performed subgroup analysis and then found that the treatment duration was the source of heterogeneity of information processing speed, but not for the other three outcome measures. A longer treatment duration can exert more significantly efficacy on information processing speed. The results of subgroup analysis showed that the different classifications of participants were the source of heterogeneity of global cognitive function, but not for the other three outcome measures. We can conclude that elderly adults without cognitive impairment are more sensitive to vitamin B supplements on global cognitive function. In spite of this, the different results of subgroup analysis may also be caused by the different characteristics and aspects of outcome measures.

There are two other studies met the inclusion criteria, but we did not include them in the meta-analysis due to the unavailability of requisite data in their published results. The study conducted by Christopher B. Brady et al. [53] reported that high daily doses of B vitamins did not affect cognitive function in patients with chronic kidney disease and end-stage renal disease. Similarly, another study conducted by Jae Hee Kang et al. [54] found that there no significant effects in cognitive function between vitamin B group and placebo group for the patients with cardiovascular disease (CVD) or CVD risk factors.

### Comparison with other studies

This systematic review revealed that vitamin B supplements can significantly lower the levels of serum Hcy and prevent the decline of global cognitive function, but was invalid for the information processing speed, episodic memory, executive function. Conversely, another two systematic reviews conducted by Andrew H. Ford et al. (2019) [22] and Hankey GJ et al. (2013) [26] respectively, reported that B-vitamin supplementation did not show an improvement in MMSE scores for individuals without cognitive impairment compared to placebo. It should be noted that MMSE was the only outcome measure in these two systematic reviews mentioned above, and yet nine different cognitive function scales were utilized to assess the global cognitive function in our systematic review. Further, a meta-analysis conducted by Clarke R et al. (2014) [25] showed that B vitamins had no significant efficacy, neither on MMSE-type global cognitive function tests nor on specific cognitive domain category (memory, speed, executive function and domain-composite global cognitive function), in spite of the Hcy lowering by dietary supplementation with B vitamins. Similarly, another two systematic reviews conducted by Annika Behrens et al. (2020) [55] and Andrew H. Ford et al. (2012) [24] show that supplementation of vitamins B12, B6, and folic acid alone or in combination does not appear to improve cognitive function in individuals without existing cognitive impairment. These two systematic reviews focused on the cognitively unimpaired individuals and they ignored the MCI patients who were in the transitional stage between normal cognitive aging and AD. Preventing or delaying the progression of MCI to AD is equally crucial. In a systematic review (2014) [23] which pooled only 2 RCTs showed that there were moderate beneficial effects of vitamins B supplementation on memory, but there were no significant difference on general cognitive function, executive function and attention in MCI patients. There was another meta-analysis conducted by Zhang et al. (2020) [56] examine the association between intake levels of vitamins B12, B6, and folate and cognitive function in older populations and found that vitamin B12, folate and vitamin B6 showed no significant benefit on cognition. The most obvious difference between this review and ours was the difference types of study design they based on. This review based on cohort studies while our systematic review based on RCTs.

### Limitations

This systematic review has several limitations worth mentioning. First, the language of the studies was restricted to English; second, the heterogeneity of studies included was substantial, so the results of this systematic review should be interpreted with caution. The conclusions drawn from the results may be susceptible to change as more homogeneous and well-designed RCTs were included in the future; third, there were four outcome measures for the evaluation of preventive efficacy in our meta-analysis, but the results of these were inconsistent. It was difficult to draw definite clinical practice recommendations in our meta-analysis.

## Conclusions and recommendations

According to the results of meta-analysis, we can draw the conclusions that vitamin B supplements can low the serum Hcy levels and improve the global cognitive function, but cannot improve the information processing speed, episodic memory, executive function of MCI patients and elderly adults without cognitive impairment. On the whole, the effect sizes among vitamin B supplements appear to be trivial. Prevention measures are never likely to exert remarkable effect sizes on individual. But for the population level, the trivial effect sizes can be amplified and result in substantial improvement.

In view of the results of different cognitive domain categories and serum Hcy, we thought that vitamin B supplements might delay or maintain the cognitive decline of elderly adults. In consideration of the vitamin B supplements is cheap and accessible, the vitamin B supplements should be considered as a preventive medicine to MCI patients or elderly adults without cognitive impairment. More well-designed RCTs with large sample sizes were required to clarify the preventive efficacy in the future. In order to obtain smaller heterogeneity, some consistency and universal cognitive function scales are necessary to evaluate the cognitive function. Moreover, we can conduct a network meta-analysis to identify the optimal dosage and combination of vitamin B12, vitamin B6, or folic acid to the cognitive decline of MCI patients and elderly adults without cognitive impairment for future work plan.

## Supplementary information

Additional file 1. The PRISMA checklist of current meta-analysis.

Additional file 2 Details of Search Strategy.

Additional file 3. Cognitive Domain Category.

Additional file 4. Excluded Studies and Reason for Exclusion.

Additional file 5. The Cochrane Risk of Bias Assessment.

## Data Availability

All data generated or analyzed during this study are included in this published article.

## Abbreviations

AD: Alzheimer’s disease
Hcy: Homocysteine
CENTRAL: The Cochrane Central Register of Controlled Trials
RCTs: Randomized controlled trials
MCI: Mild cognitive impairment
SMD: Standardized mean difference
MD: Mean difference
CI: Confidence interval
ADI: Alzheimer’s Disease International
Aβ: β-amyloid peptide
NFTs: neurofibrillary tangles
MMSE: Mini-Mental State Examination
PRISMA: Preferred Reporting Items for Systematic Reviews and Meta-Analyses
MESH: Medical subject headings
ITT: Intention-to-treat
SD_*change*_: SD of mean change
Corr: Correlation coefficient
SD_*baseline*_: SD of baseline
SD_*final*_: SD of final
SI: Systeme International
IG: Intervention group
CG: Control group
NR: Not reported
EAWCI: Elderly adults without cognitive impairment
NA: Not applicable

## References

[1] Lane CA, Hardy J, Schott JM (2018). Alzheimer’s disease. Eur J Neurol.

[2] Bhatt J, Comas Herrera A, Amico F, Farina N, Wong J, Orange J (2019). The World Alzheimer Report 2019: Attitudes to dementia.

[3] Pyenson B, Sawhney TG, Steffens C, Rotter D, Peschin S, Scott J (2019). The Real-World Medicare Costs of Alzheimer Disease: Considerations for Policy and Care. J Manag Care Spec Pharm.

[4] Marešová P, Dolejs J, Mohelska H, Bryan LK (2019). Cost of Treatment and Care for People with Alzheimer’s Disease: A Meta- Analysis. Curr Alzheimer Res.

[5] Michaud TL, Su D, Siahpush M, Murman DL (2017). The Risk of Incident Mild Cognitive Impairment and Progression to Dementia Considering Mild Cognitive Impairment Subtypes. Dement Geriatr Cogn Dis Extra.

[6] Petersen RC, Doody R, Kurz A, Mohs RC, Morris JC, Rabins PV (2001). Current concepts in mild cognitive impairment. Arch Neurol.

[7] Lee HK, Kim SY, Sok SR (2016). Effects of Multivitamin Supplements on Cognitive Function, Serum Homocysteine Level, and Depression of Korean Older Adults With Mild Cognitive Impairment in Care Facilities. J Nurs Scholarsh.

[8] Pal K, Mukadam N, Petersen I, Cooper C (2018). Mild cognitive impairment and progression to dementia in people with diabetes, prediabetes and metabolic syndrome: a systematic review and meta-analysis. Soc Psychiatry Psychiatr Epidemiol.

[9] Sperling RA, Aisen PS, Beckett LA, Bennett DA, Craft S, Fagan AM (2011). Toward defining the preclinical stages of Alzheimer’s disease: recommendations from the National Institute on Aging-Alzheimer’s Association workgroups on diagnostic guidelines for Alzheimer’s disease. Alzheimers Dement.

[10] O’Brien RJ, Wong PC (2011). Amyloid Precursor Protein Processing and Alzheimer’s Disease. Annu Rev Neurosci.

[11] Takahashi RH, Nagao T, Gouras GK (2017). Plaque formation and the intraneuronal accumulation of β-amyloid in Alzheimer’s disease. Pathol Int.

[12] Ho PI, Collins SC, Dhitavat S, Ortiz D, Ashline D, Rogers E (2001). Homocysteine potentiates beta-amyloid neurotoxicity: role of oxidative stress. J Neurochem.

[13] Vogel T, Dali-Youcef N, Kaltenbach G, Andres E (2009). Homocysteine, vitamin B12, folate and cognitive functions: a systematic and critical review of the literature. Int J Clin Pract.

[14] Porter K, Hoey L, Hughes CF, Ward M, McNulty H (2016). Causes, Consequences and Public Health Implications of Low B-Vitamin Status in Ageing. Nutrients.

[15] Morris MS (2012). The role of B vitamins in preventing and treating cognitive impairment and decline. Adv Nutr.

[16] Douaud G, Refsum H, de Jager CA, Jacoby R, Nichols TE, Smith SM (2013). Preventing Alzheimer’s disease-related gray matter atrophy by B-vitamin treatment. Proc Natl Acad Sci U S A.

[17] Collaboration HLT (2005). Dose-dependent effects of folic acid on blood concentrations of homocysteine: a meta-analysis of the randomized trials. Am J Clin Nutr.

[18] Elsherbiny NM, Sharma I, Kira D, Alhusban S, Samra YA, Jadeja R (2020). Homocysteine Induces Inflammation in Retina and Brain. Biomolecules.

[19] Moore K, Hughes CF, Ward M, Hoey L, McNulty H (2018). Diet, nutrition and the ageing brain. current evidence and new directions. Proc Nutr Soc.

[20] Kane RL, Butler M, Fink HA, Brasure M, Davila H, Desai P, et al. Interventions to Prevent Age-Related Cognitive Decline, Mild Cognitive Impairment, and Clinical Alzheimer’s-Type Dementia. Rockville (MD). Agency for Healthcare Research and Quality (US); 2017. 28759193

[21] Enderami A, Zarghami M, Darvishi-Khezri H (2018). The effects and potential mechanisms of folic acid on cognitive function: a comprehensive review. Neu Sci.

[22] Ford AH, Almeida OP (2019). Effect of Vitamin B Supplementation on Cognitive Function in the Elderly: A Systematic Review and Meta-Analysis. Drugs Aging.

[23] Li MM, Yu JT, Wang HF, Jiang T, Wang J, Meng XF (2014). Efficacy of Vitamins B Supplementation on Mild Cognitive Impairment and Alzheimer’s Disease: A Systematic Review and Meta-Analysis. Curr Alzheimer Res.

[24] Ford AH, Almeida OP (2012). Effect of homocysteine lowering treatment on cognitive function: a systematic review and meta-analysis of randomized controlled trials. J Alzheimers Dis.

[25] Clarke R, Bennett D, Parish S, Lewington S, Skeaff M, Eussen SJ (2014). Effects of homocysteine lowering with B vitamins on cognitive aging: meta-analysis of 11 trials with cognitive data on 22,000 individuals. Am J Clin Nutr.

[26] Hankey GJ, Ford AH, Yi Q, Eikelboom JW, Lees KR, Chen C (2013). Effect of B vitamins and lowering homocysteine on cognitive impairment in patients with previous stroke or transient ischemic attack: a prespecified secondary analysis of a randomized, placebo-controlled trial and meta-analysis. Stroke.

[27] Jia X, McNeill, Avenell A (2008). Does taking vitamin, mineral and fatty acid supplements prevent cognitive decline? A systematic review of randomized controlled trials. J Hum Nutr Diet.

[28] Ma F, Zhou X, Li Q, Zhao JG, Song AL, An PL (2019). Effects of Folic Acid and Vitamin B-12, Alone and in Combination on Cognitive Function and Inflammatory Factors in the Elderly with Mild Cognitive Impairment: A Single-blind Experimental Design. Curr Alzheimer Res.

[29] Kwok T, Wu Y, Lee J, Lee R, Yung CY, Choi G, et al. A randomized placebo-controlled trial of using B vitamins to prevent cognitive decline in older mild cognitive impairment patients. Clin Nutr. 2019;S0261-5614(19)33132-2. 10.1016/j.clnu.2019.11.005 31787369

[30] Ma F, Li Q, Zhou X, Zhao J, Song A, Li W (2017). Effects of folic acid supplementation on cognitive function and Abeta-related biomarkers in mild cognitive impairment: a randomized controlled trial. Eur J Nutr.

[31] Briggs R, Kennelly SP, O’Neill D (2016). Drug treatments in Alzheimer’s disease. Clin Med (Lond).

[32] Moher D, Shamseer L, Clarke M, Ghersi D, Liberati A, Petticrew M (2015). Preferred reporting items for systematic review and meta-analysis protocols (PRISMA-P) 2015 statement. Syst Rev.

[33] Higgins JPTTJ, Chandler J, Cumpston M, Li T, Page MJ, Welch VA, editors. Cochrane Handbook for Systematic Reviews of Interventions, 2nd Edition edn. Chichester (UK): John Wiley & Sons; 2019.

[34] Kwok T, Lee J, Ma RC, Wong SY, Kung K, Lam A (2017). A randomized placebo controlled trial of vitamin B12 supplementation to prevent cognitive decline in older diabetic people with borderline low serum vitamin B12. Clin Nutr.

[35] Cheng D, Kong H, Pang W, Yang H, Lu H, Huang C (2016). B vitamin supplementation improves cognitive function in the middle aged and elderly with hyperhomocysteinemia. Nutr Neurosci.

[36] Dangour AD, Allen E, Clarke R, Elbourne D, Fletcher AE, Letley L (2015). Effects of vitamin B-12 supplementation on neurologic and cognitive function in older people: a randomized controlled trial. Am J Clin Nutr.

[37] van der Zwaluw NL, Dhonukshe-Rutten RA, van Wijngaarden JP, Brouwer-Brolsma EM, van de Rest O, In ‘t Veld PH, et al. Results of 2-year vitamin B treatment on cognitive performance: secondary data from an RCT. Neurology. 2014;83(23):2158-66. 10.1212/WNL.0000000000001050 25391305

[38] Walker JG, Batterham PJ, Mackinnon AJ, Jorm AF, Hickie I, Fenech M (2012). Oral folic acid and vitamin B-12 supplementation to prevent cognitive decline in community-dwelling older adults with depressive symptoms–the Beyond Ageing Project: a randomized controlled trial. Am J Clin Nutr.

[39] de Jager CA, Oulhaj A, Jacoby R, Refsum H, Smith AD (2012). Cognitive and clinical outcomes of homocysteine-lowering B-vitamin treatment in mild cognitive impairment: a randomized controlled trial. Int J Geriatr Psychiatry.

[40] Ford AH, Flicker L, Alfonso H, Thomas J, Clarnette R, Martins R (2010). Vitamins B(12), B(6), and folic acid for cognition in older men. Neurology.

[41] van Uffelen JG, Chinapaw MJ, van Mechelen W, Hopman-Rock M (2008). Walking or vitamin B for cognition in older adults with mild cognitive impairment? A randomised controlled trial. Br J Sports Med.

[42] Durga J, van Boxtel MPJ, Schouten EG, Kok FJ, Jolles J, Katan MB (2007). Effect of 3-year folic acid supplementation on cognitive function in older adults in the FACIT trial: a randomised, double blind, controlled trial. Lancet.

[43] McMahon JA, Green TJ, Skeaff CM, Knight RG, Mann JI, Williams SM (2006). A controlled trial of homocysteine lowering and cognitive performance. N Engl J Med.

[44] Eussen SJ, de Groot LC, Joosten LW, Bloo RJ, Clarke R, Ueland PM (2006). Effect of oral vitamin B-12 with or without folic acid on cognitive function in older people with mild vitamin B-12 deficiency: a randomized, placebo-controlled trial. Am J Clin Nutr.

[45] Stott DJ, MacIntosh G, Lowe GD, Rumley A, McMahon AD, Langhorne P (2005). Randomized controlled trial of homocysteine-lowering vitamin treatment in elderly patients with vascular disease. Am J Clin Nutr.

[46] Lewerin C, Matousek M, Steen G, Johansson B, Steen B, Nilsson-Ehle H (2005). Significant correlations of plasma homocysteine and serum methylmalonic acid with movement and cognitive performance in elderly subjects but no improvement from short-term vitamin therapy: a placebo-controlled randomized study. Am J Clin Nutr.

[47] Hvas A-M, Juul S, Lauritzen L, Nexø E, Ellegaard J (2004). No effect of vitamin B-12 treatment on cognitive function and depression: a randomized placebo controlled study. J Affect Disord.

[48] Angeles G, Kate P, Katherine Z, Andrew D (2004). Cobalamin Reduces Homocysteine in Older Adults on Folic Acid-Fortified Diet: A Pilot, Double-Blind, Randomized, Placebo-Controlled Trial. J Am Geriatr Soc.

[49] Bryan J, Calvaresi E, Hughes D (2002). Short-term folate, vitamin B-12 or vitamin B-6 supplementation slightly affects memory performance but not mood in women of various ages. J Nutr.

[50] Fioravanti M, Ferrario E, Massaia M, Cappa G, Rivolta G, Grossi E (1998). Low folate levels in the cognitive decline of elderly patients and the efficacy of folate as a treatment for improving memory deficits. Arch Gerontol Geriatr.

[51] Higgins JPTSJ, Page MJ, Elbers RG, Sterne JAC. Chapter 8: Assessing risk of bias in a randomized trial. In: Higgins JPT, Thomas J, Chandler J, Cumpston M, Li T, Page MJ, Welch VA, editors. Cochrane Handbook for Systematic Reviews of Interventions version 6.0 (updated July 2019). Cochrane; 2019.

[52] Smith AD, Smith SM, de Jager CA, Whitbread P, Johnston C, Agacinski G (2010). Homocysteine-lowering by B vitamins slows the rate of accelerated brain atrophy in mild cognitive impairment: a randomized controlled trial. PloS one.

[53] Brady CB, Gaziano JM, Cxypoliski RA, Guarino PD, Kaufman JS, Warren SR (2009). Homocysteine lowering and cognition in CKD: the Veterans Affairs homocysteine study. Am J Kidney Dis.

[54] Kang JH, Cook N, Manson J, Buring JE, Albert CM, Grodstein F (2008). A trial of B vitamins and cognitive function among women at high risk of cardiovascular disease. Am J Clin Nutr.

[55] Behrens A, Graessel E, Pendergrass A, Donath C (2020). Vitamin B-Can it prevent cognitive decline? A systematic review and meta-analysis. Syst Rev.

[56] Zhang C, Luo J, Yuan C, Ding D (2020). Vitamin B12, B6, or Folate and Cognitive Function in Community-Dwelling Older Adults: A Systematic Review and Meta-Analysis. J Alzheimers Dis.

