# Supplementary material for: The preventive efficacy of vitamin B supplements on the cognitive decline of elderly adults: a systematic review and meta-analysis
Source: BMC Geriatr. 2021 Jun 16;21:367. doi: 10.1186/s12877-021-02253-3 (PMC8207668; doi:10.1186/s12877-021-02253-3)
Supplement: Supplementary file 2 — Details of Search Strategy. [file 12877_2021_2253_MOESM2_ESM.docx]

# Additional file 2: Details of Search Strategy

**1.PubMed** **(retrieved in December 1, 2019 for the first time and update in June 1, 2020)**

| Items | Search Terms | Search Results |
| --- | --- | --- |
| #1 (participants) | ((((((((((((((((((((((((Cognitive Dysfunction[MeSH Terms]) OR MCI[Title/Abstract]) OR mild cognitive impairment[Title/Abstract]) OR Aged[MeSH Terms]) OR elderly[Title/Abstract]) OR older[Title/Abstract]) OR geriatric[Title/Abstract]) OR aging[Title/Abstract]) OR aged[Title/Abstract]) OR seniors[Title/Abstract]) OR senior citizens[Title/Abstract]) OR community dwelling[Title/Abstract]) OR aged population[Title/Abstract]) OR pensioners[Title/Abstract]) OR Memory Disorders[MeSH Terms]) OR episodic memory[Title/Abstract]) OR mental disorder[Title/Abstract]) OR cognitive decline[Title/Abstract]) OR cognition reduce[Title/Abstract]) OR cognitive disorder[Title/Abstract]) OR cognitive impairment[Title/Abstract]) OR cognitive function[Title/Abstract]) OR cognition function[Title/Abstract]) OR cognitive performance[Title/Abstract]) OR cognitive deficit[Title/Abstract] | 3907016 |
| #2 (intervention) | (((((((((((((((((("Vitamin B 12"[Mesh]) OR Vitamin B 6[MeSH Terms]) OR folic acid[MeSH Terms]) OR B12, Vitamin[Title/Abstract]) OR Vitamin B 12[Title/Abstract]) OR cobalamin[Title/Abstract]) OR cobalamins[Title/Abstract]) OR cyanocobalamin[Title/Abstract]) OR vitamin B 6[Title/Abstract]) OR vitamin B6[Title/Abstract]) OR pyridoxine[Title/Abstract]) OR pyridoxal[Title/Abstract]) OR pyridoxamine[Title/Abstract]) OR folic acid[Title/Abstract]) OR folate[Title/Abstract]) OR B9,vitamin[Title]) OR vitamin B9[Title/Abstract]) OR folacin[Title/Abstract]) | 95507 |
| #3 (study) | ((clinical [Title/Abstract] AND trial [Title/Abstract]) OR clinical trials as topic [MeSH Terms] OR clinical trial [Publication Type] OR random*[Title/Abstract] OR random allocation [MeSH Terms] OR therapeutic use [MeSH Subheading]) | 5484449 |
| #4 | (rat [Title/Abstract]) or (mouse [Title/Abstract]) | 1436307 |
| #5 | #1 and #2 and #3 not #4 | 11248 |

**2.Embase (retrieved in December 1, 2019 for the first time and update in June 1, 2020)**

| Items | Search Terms | Search Results |
| --- | --- | --- |
| #1 (participants) | ('mild cognitive impairment'/exp OR mci:ti,ab OR 'cognitive defect'/exp OR 'cognitive dysfunction':ti,ab OR 'aged'/exp OR elderly:ti,ab OR 'older adults'/exp OR older:ti,ab OR 'geriatrics'/exp OR 'aging'/exp OR 'senior citizens':ti,ab OR 'community dwelling person'/exp OR 'community dwelling individuals' OR 'community dwelling participants' OR 'community dwelling people' OR 'community dwelling person' OR 'community dwelling persons' OR 'community dwelling subjects' OR 'community-dwelling cohort' OR 'community dwelling':ti,ab OR 'aged population':ti,ab OR 'pensioners':ti,ab OR 'memory disorder'/exp OR 'cognitive decline':ti,ab OR 'cognition reduce':ti,ab OR 'cognitive  disorder':ti,ab OR 'cognitive impairment':ti,ab OR 'cognitive function':ti,ab OR 'cognition function':ti,ab OR 'cognitive performance':ti,ab OR 'cognitive deficit':ti,ab) | 4083731 |
| #2 (intervention) | ('cyanocobalamin'/exp OR 'pyridoxine'/exp OR 'folic acid'/exp OR 'vitamin b 12':ti,ab OR 'cobalamin':ti,ab OR cobalamins:ti,ab OR cyanocobalamin:ti,ab OR 'vitamin b 6':ti,ab OR pyridoxine:ti,ab OR pyridoxal:ti,ab OR pyridoxamine:ti,ab OR 'folic acid':ti,ab OR folate:ti,ab OR 'vitamin b 9':ti,ab OR folacin:ti,ab) | 131454 |
| #3 (study) | ('randomized controlled trial'/exp OR 'randomized controlled trial':ti,ab OR 'randomization'/exp OR 'random allocation':ti,ab OR 'placebo'/exp OR 'controlled clinical trial'/exp OR randomized:ti,ab OR 'controlled clinical trial':ti,ab OR 'double blind procedure'/exp OR 'single blind procedure'/exp OR 'multicenter study'/exp OR 'double blind procedure':ti,ab OR 'single blind procedure':ti,ab OR 'multicenter study':ti,ab) | 1572707 |
| #4 | #1 and #2 and #3 | 2497 |

**3.The Cochrane library (From inception to December 1, 2019)**

| Items | Search Terms | Search Results |
| --- | --- | --- |
| #1 (participants) | (cognition function): ti,ab,kw OR (Memory Disorders): ti,ab,kw OR (cognitive decline): ti,ab,kw OR (MCI): ti,ab,kw OR (cognitive deficit): ti,ab,kw OR (elderly): ti,ab,kw OR (older): ti,ab,kw OR (aging): ti,ab,kw OR (aged): ti,ab,kw OR (community dweling): ti,ab,kw OR MeSH descriptor: [Cognitive Dysfunction] explode all trees OR MeSH descriptor: [Memory Disorders] explode all trees OR MeSH descriptor: [Aged] explode all trees | 695207 |
| #2 (intervention) | (Vitamin B12): ti,ab,kw OR (cobalamin): ti,ab,kw OR (cyanocobalamin): ti,ab,kw OR (Vitamin B6): ti,ab,kw OR (pyridoxine): ti,ab,kw OR (folic acid): ti,ab,kw OR (folate): ti,ab,kw OR (Vitamin B9): ti,ab,kw OR (folacin): ti,ab,kw OR (pyridoxal): ti,ab,kw OR MeSH descriptor: [Vitamin B 12] explode all trees OR MeSH descriptor: [Vitamin B 6] explode all trees OR MeSH descriptor: [Folic Acid] explode all trees | 8782 |
| #3 (study) | (clinical trial): ti,ab,kw OR (randomized controlled trial): ti,ab,kw OR (randomization): ti,ab,kw OR (random allocation): ti,ab,kw OR (placebo): ti,ab,kw OR (double blind): ti,ab,kw OR (single blind): ti,ab,kw OR MeSH descriptor: [Randomized Controlled Trial] explode all trees OR MeSH descriptor: [Random Allocation] explode all trees OR MeSH descriptor: [Clinical Trial] explode all trees | 1146191 |
| #4 | #1 and #2 and #3 | 4450 |

**4.** **Web of Science (retrieved in December 1, 2019 for the first time and update in June 1, 2020)**

| Items | Search Terms | Search Results |
| --- | --- | --- |
| #1 (participants) | TS= (cognitive deficit or cognitive performance or cognition function or cognitive function or cognitive impairment or cognitive disorder or cognition reduce or cognitive decline or mental disorder or Memory Disorders or pensioners or aged population or community dwelling or senior citizens or seniors or aged or aging or geriatric or older or elderly or mild cognitive impairment or Cognitive Dysfunction) | 4171096 |
| #2 (intervention) | TS= (vitamin B 12 or vitamin B 6 or folic acid or cobalamin or cobalamins or cyanocobalamin or pyridoxine or pyridoxal or pyridoxamine or folate or vitamin B 9 OR folacin) | 84563 |
| #3 (study) | TS= (randomized controlled trial or controlled clinical trial or randomized or placebo or randomly or multicenter study) | 1336718 |
| #4 | #1 and #2 and #3 | 2680 |

**5.** Science Direct **(retrieved in December 1, 2019 for the first time and update in June 1, 2020)**

| Items | Search Terms | Search Results |
| --- | --- | --- |
| #1 (participants) | mild cognitive impairment OR cognitive dysfunction OR aged OR older | 1125553 |
| #2 (intervention) | Vitamin B 12 OR vitamin B 6 OR folic acid | 10566 |
| #3 (study) | randomized OR randomly | 408559 |
| #4 | #1 and #2 and #3 | 231 |

**6. Scopus (retrieved in December 1, 2019 for the first time and update in June 1, 2020)**

| Items | Search Terms | Search Results |
| --- | --- | --- |
| #1 (participants) | TITLE-ABS-KEY ("cognitive dysfunction" OR "mild cognitive impairment" OR aged OR elderly OR older OR geriatric OR aging OR "senior citizens" OR "community dwelling" OR "mental disorder" OR "cognitive decline" OR "cognition reduce" OR "cognitive function" OR "cognitive performance" OR "cognitive deficit") | 7616322 |
| #2(intervention) | TITLE-ABS-KEY ("vitamin B 12" OR "vitamin B 6" OR "folic acid" OR "cobalamin" OR "cobalamins" OR "cyanocobalamin" OR "pyridoxine" OR "pyridoxal" OR "pyridoxamine" OR "folate" OR "vitamin B 9") | 145610 |
| #3(study) | TITLE-ABS-KEY ("randomized controlled trial" OR "controlled clinical trial" OR "randomized" OR "placebo" OR "randomly") | 1796837 |
| #4 | #1 and #2 and #3 | 4361 |

**7.PsycINFO (retrieved in December 1, 2019 for the first time and update in June 1, 2020)**

| Items | Search Terms | Search Results |
| --- | --- | --- |
| #1 (participants) | Abstract: Cognitive Dysfunction OR Abstract: mild cognitive impairment OR Abstract: elderly OR Abstract: aging OR Abstract: cognitive decline OR Abstract: MCI OR Abstract: older OR Abstract: aged OR Abstract: seniors OR Abstract: community dwelling OR Abstract: pensioners OR Abstract: Memory Disorders OR Abstract: mental disorder OR Abstract: cognitive disorder OR Abstract: cognitive function OR Abstract: cognition function OR Abstract: cognitive deficit | 543103 |
| #2 (intervention) | Abstract: Vitamin B12 OR Abstract: Vitamin B6 OR Abstract: folic acid OR Abstract: cobalamin OR Abstract: cobalamins OR Abstract: cyanocobalamin OR Abstract: pyridoxine OR Abstract: pyridoxal OR Abstract: pyridoxamine OR Abstract: folate OR Abstract: Vitamin B9 OR Abstract: folacin | 2618 |
| #3 (study) | Abstract: clinical trial OR Abstract: random OR Abstract: random allocation OR Abstract: randomized controlled trial OR Abstract: randomized OR Abstract: placebo OR Abstract: randomly OR Abstract: double blind OR Abstract: randomization | 214199 |
| #4 | #1 and #2 and #3 | 138 |
