# Supplementary material for: The preventive efficacy of vitamin B supplements on the cognitive decline of elderly adults: a systematic review and meta-analysis
Source: BMC Geriatr. 2021 Jun 16;21:367. doi: 10.1186/s12877-021-02253-3 (PMC8207668; doi:10.1186/s12877-021-02253-3)
Supplement: Supplementary file 3 — Cognitive Domain Category. [file 12877_2021_2253_MOESM3_ESM.docx]

# Additional file 3: Cognitive Domain Category

| **First Author**  **(Year) Country** | **global cognitive function** | **information processing speed** | **executive function** | **memory** |
| --- | --- | --- | --- | --- |
| Fei Ma (2019) China | Full Scale IQ (FSIQ) | - | - | - |
| Kwok Timothy (2019) China | Clinical dementia rating scale sum of boxes (CDR_SOB) | - | Controlled Oral Word Association Test (COWAT);  Category Fluency Test (CFT) | Continuous Paired Associates Learning (CPAL);  International Shopping List Test (ISLT) |
| Fei Ma (2017) China | Full Scale IQ (FSIQ) | - | - | - |
| Kwok Timothy (2017) China | Neuropsychological Test Battery (NTB) | Simple Reaction Time (SRT);  Choice Reaction Time test (CRT) | Controlled Oral Word Association Test (COWAT);  Category Fluency Test (CFT) | Continuous Paired Associates Learning (CPAL);  International Shopping List Test (ISLT) |
| Cheng D (2016) China | Basic Cognitive Aptitude Tests (BCATs) | - | - | - |
| Dangour AD (2015)  United Kingdom | - | Symbol letter modality | Verbal fluency test | California Verbal Learning Test (CVLT) |
| van der Zwaluw NL (2014) Netherlands | Mini-Mental State Examination (MMSE) | Trail Making part A;  Stroop 1 and 2 mean; Symbol Digit Modalities Test | Trail Making Test;  Stroop Interference;  Verbal Fluency–total | Rey Auditory Verbal Learning Test (RAVLT)–Immediate Recall;  Rey Auditory Verbal Learning Test (RAVLT) -Decay;  Rey Auditory Verbal Learning Test (RAVLT)–Recognition |
| Hankey GJ (2013) Australia | Mini-Mental State Examination (MMSE) | - |  | - |
| Walker JG (2012) Australia | Telephone Interview for Cognitive Status-Modified (TICS-M) | - | - | - |
| de Jager CA (2012) United Kingdom | Clinical dementia rating scale sum of boxes (CDR_SOB); Mini-Mental State Examination (MMSE) |  | Executive Clock Drawing Task (CLOX) | Hopkins Verbal Learning Test–delayed recall (HVLT DR); category fluency |
| Ford AH (2010) Australia | Alzheimer’s Disease Assessment Scale (ADAS-cog);  Mini-Mental State Examination (MMSE) | - | - | California Verbal Learning Test List A immediate free recall trials 1–5 total (CVLT1);  California Verbal Learning Test List A long-delay free recall (CVLT2) |
| van Uffelen JG (2008) Netherlands | Mini-Mental State Examination (MMSE) | digit symbol substitution  test (DSST) | verbal fluency test (VFT) | auditory verbal learning test immediate and delayed recall (AVLT1-5);  auditory verbal learning test maximum scores 75 and 15  words (AVLT6) |
| Durga J (2007) Netherlands | Global cognitive function | Letter Digit Substitution test | Verbal Fluency test (VFT) | 15-Word Learning test |
| McMahon JA (2006) New Zealand | Mini-Mental State Examination (MMSE) | Part B of the Reitan Trail Making Test | Category Word Fluency test;  Raven’s Progressive Matrices;  Controlled Oral Word Association Test | Wechsler Paragraph Recall test;  Rey Auditory Verbal Learning Test (RAVLT) |
| Eussen SJ (2006) Netherlands | - | Motor planning 2;  Finger tapping;  Trail making test | Motor planning 3;  Trail making test;  Stroop test;  Similarities WAIS;  Raven;  Word fluency | 15 Word learning;  Complex figure of Rey;  Digit span backward |
| Stott DJ (2005)  United Kingdom | Telephone Interview for Cognitive Status-Modified (TICS-M) | Letter Digit Coding Test (LDCT) | - | - |
| Lewerin C (2005) Sweden | - | Identical forms;  Digit symbol | Synonyms;  Block design;  Figure classification | Digit span forward;  Digit span backward;  Visual reproduction;  Thurstone’s picture memory |
| Hvas AM (2004) Denmark | Cambridge Cognitive  Examination (CAMCOG);  Mini-Mental State Examination (MMSE) | - | - | 12 words learning test |
| Garcia A (2004) Canada | Mini-Mental State Examination (MMSE);  Clinical dementia rating (CDR) | - | - | California Verbal Learning Test Trial A Lists 1–5 score (CVLT) |
| Janet Bryan (2002)  Australis |  | The Boxes Test; Digit-Symbol Coding; Symbol Search subtests of the Wechsler Adult Intelligence Scale-III | Stroop Test; Self-Ordered Pointing Task; Uses for Common Objects; Trail Making Test; Verbal Fluency, comprising Initial Letter Fluency; Excluded Letter Fluency | The Rey Auditory-Verbal Learning Test (RAVLT); Digit-Symbol-Coding (WAIS-III); Activity recall. |
| Fioravanti M (1997) Italy | - | - | - | Acquisition and recall;  Delayed call;  Memory index;  Encoding factor;  Cognitive efficiency |
