# Supplementary material for: The preventive efficacy of vitamin B supplements on the cognitive decline of elderly adults: a systematic review and meta-analysis
Source: BMC Geriatr. 2021 Jun 16;21:367. doi: 10.1186/s12877-021-02253-3 (PMC8207668; doi:10.1186/s12877-021-02253-3)
Supplement: Supplementary file 4 — Excluded Studies and Reason for Exclusion. [file 12877_2021_2253_MOESM4_ESM.docx]

# Additional file 4: Excluded Studies and Reason for Exclusion

| N | First Author（Year） | Title | Reason for exclusion |
| --- | --- | --- | --- |
| 1 | Lee HK (2016) [1] | Effects of Multivitamin Supplements on Cognitive Function, Serum Homocysteine Level, and Depression of Korean Older Adults with Mild Cognitive Impairment in Care Facilities | It’s a pretest-posttest control group design, which utilized convenience sampling |
| 2 | Ma F (2016) [2] | Folic acid supplementation improves cognitive function by reducing the levels of peripheral inflammatory cytokines in elderly Chinese subjects with MCI | The data was extracted from intermediate process of a long-term trial which had been included in our meta-analysis |
| 3 | Ma F (2016) [3] | Effects of 6-Month Folic Acid Supplementation on Cognitive Function and Blood Biomarkers in Mild Cognitive Impairment: A Randomized Controlled Trial in China | The data was extracted from intermediate process of a long-term trial which had been included in our meta-analysis |
| 4 | Douaud G (2013) [4] | Preventing Alzheimer's disease-related gray matter atrophy by B-vitamin treatment | No applicable outcome measures of cognitive function |
| 5 | Andreeva VA (2011) [5] | Cognitive function after supplementation with B vitamins and long-chain omega-3 fatty acids: ancillary findings from the SU.FOL.OM3 randomized trial | Ineligible population: the age of participants was 45~80 years; no baseline data of the outcome measures |
| 6 | Smith AD (2010) [6] | Homocysteine-lowering by B vitamins slows the rate of accelerated brain atrophy in mild cognitive impairment: a randomized controlled trial | No applicable outcome measures of cognitive function |
| 7 | Armitage JM (2010) [7] | Effects of homocysteine-lowering with folic acid plus vitamin B12 vs placebo on mortality and major morbidity in myocardial infarction survivors: a randomized trial | Ineligible population: the age of participants was 18~80 years; no applicable outcome measures of cognitive function |
| 8 | Brady CB (2009) [8] | Homocysteine lowering and cognition in CKD: The Veterans Affairs homocysteine study | Valid data of outcome measure was unavailable |
| 9 | Kang JH (2008) [9] | A trial of B vitamins and cognitive function among women at high risk of cardiovascular disease | Valid data of outcome measure was unavailable |
| 10 | Pathansali Rohan (2006) [10] | Effects of folic acid supplementation on psychomotor performance and hemorheology in healthy elderly subjects | No applicable outcome measures of cognitive function |
| 11 | Obeid R (2005) [11] | Effect of B vitamins on cognitive function in elderly people with mild cognitive dysfunction | Conference paper which full-text was not found |
| 12 | Clarke R (2003) [12] | Effect of vitamins and aspirin on markers of platelet activation, oxidative stress and homocysteine in people at high risk of dementia | Ineligible population: elderly adults with dementia or mild cognitive  impairment |
| 13 | Seal EC (2002) [13] | A randomized, double-blind, placebo-controlled study of oral vitamin B12 supplementation in older patients with subnormal or borderline serum vitamin B12 concentrations | Ineligible population: some of the elderly adults with dementia |
| 14 | Henning BF (2001) [14] | Long-Term Effects of Vitamin B12, Folate, and Vitamin B6 Supplements in Elderly People with Normal Serum Vitamin B12 Concentrations | No applicable outcome measures of cognitive function |
| 15 | Kwok T (1998) [15] | Randomized trial of the effect of supplementation on the cognitive function of older people with subnormal cobalamin levels | Ineligible population: elderly adults with dementia or without dementia |
| 16 | La Rue A (1997) [16] | Nutritional status and cognitive functioning in a normally aging sample: a 6-y reassessment | Not a RCT |
| 17 | Naurath HJ (1995) [17] | Effects of vitamin B12, folate, and vitamin B6 supplements in elderly people with normal serum vitamin concentrations | No applicable outcome measures of cognitive function |
| 18 | Deijen JB (1992) [18] | Vitamin B-6 supplementation in elderly men: effects on mood, memory, performance and mental effort | No applicable outcome measures of cognitive function |

Reference:

1. Lee HK, Kim SY, Sok SR. Effects of Multivitamin Supplements on Cognitive Function, Serum Homocysteine Level, and Depression of Korean Older Adults With Mild Cognitive Impairment in Care Facilities. Journal of Nursing Scholarship. 2016;48(3):223-31.

2. Ma F, Wu T, Zhao J, Song A, Liu H, Xu W, Huang G. Folic acid supplementation improves cognitive function by reducing the levels of peripheral inflammatory cytokines in elderly Chinese subjects with MCI. Scientific reports. 2016;6:37486.

3. Ma F, Wu T, Zhao J, Han F, Marseglia A, Liu H, Huang G. Effects of 6-Month Folic Acid Supplementation on Cognitive Function and Blood Biomarkers in Mild Cognitive Impairment: A Randomized Controlled Trial in China. The journals of gerontology Series A, Biological sciences and medical sciences. 2016;71(10):1376-83.

4. Douaud G, Refsum H, de Jager CA, Jacoby R, Nichols TE, Smith SM, Smith AD. Preventing Alzheimer's disease-related gray matter atrophy by B-vitamin treatment. Proceedings of the National Academy of Sciences of the United States of America. 2013;110(23):9523-8.

5. Andreeva VA, Kesse-Guyot E, Barberger-Gateau P, Fezeu L, Hercberg S, Galan P. Cognitive function after supplementation with B vitamins and long-chain omega-3 fatty acids: ancillary findings from the SU.FOL.OM3 randomized trial. The American journal of clinical nutrition. 2011;94(1):278-86.

6. Smith AD, Smith SM, de Jager CA, Whitbread P, Johnston C, Agacinski G, Oulhaj A, Bradley KM, Jacoby R, Refsum H. Homocysteine-lowering by B vitamins slows the rate of accelerated brain atrophy in mild cognitive impairment: a randomized controlled trial. PloS one. 2010;5(9):e12244.

7. Study of the Effectiveness of Additional Reductions in C, Homocysteine Collaborative G, Armitage JM, Bowman L, Clarke RJ, Wallendszus K, Bulbulia R, Rahimi K, Haynes R, Parish S et al. Effects of homocysteine-lowering with folic acid plus vitamin B12 vs placebo on mortality and major morbidity in myocardial infarction survivors: a randomized trial. Jama. 2010;303(24):2486-94.

8. Brady CB, Gaziano JM, Cxypoliski RA, Guarino PD, Kaufman JS, Warren SR, Hartigan P, Goldfarb DS, Jamison RL. Homocysteine lowering and cognition in CKD: the Veterans Affairs homocysteine study. Am J Kidney Dis. 2009;54(3):440-9.

9. Kang JH, Cook N, Manson J, Buring JE, Albert CM, Grodstein F. A trial of B vitamins and cognitive function among women at high risk of cardiovascular disease. The American journal of clinical nutrition. 2008;88(6):1602-10.

10. Pathansali R, Mangoni AA, Creagh-Brown B, Lan ZC, Ngow GL, Yuan XF, Ouldred EL, Sherwood RA, Swift CG, Jackson SH. Effects of folic acid supplementation on psychomotor performance and hemorheology in healthy elderly subjects. Archives of gerontology and geriatrics. 2006;43(1):127-37.

11. Obeid R, Fink-Geisel U, Eckert R, Herrmann W. Effect of B vitamins on cognitive function in elderly people with mild cognitive dysfunction. European Congress of Clinical Biochemistry and Laboratory Medicine; 2005.

12. Clarke R, Harrison G Fau - Richards S, Richards S. Effect of vitamins and aspirin on markers of platelet activation, oxidative stress and homocysteine in people at high risk of dementia. J Intern Med. 2003;254(1):67-75.

13. Seal EC, Metz J, Flicker L, Melny J. A randomized, double-blind, placebo-controlled study of oral vitamin B12 supplementation in older patients with subnormal or borderline serum vitamin B12 concentrations. J Am Geriatr Soc. 2002;50(1):146-51.

14. Henning BF, Tepel M Fau - Riezler R, Riezler R Fau - Naurath HJ, Naurath HJ. Long-Term Effects of Vitamin B12, Folate, and Vitamin B6 Supplements in Elderly People with Normal Serum Vitamin B12 Concentrations. Gerontology. 2001;47(1):30-5.

15. Kwok T, Tang C, Woo J, Lai WK, Law LK, Pang CP. Randomized trial of the effect of supplementation on the cognitive function of older people with subnormal cobalamin levels. International journal of geriatric psychiatry. 1998;13(9):611-6.

16. La Rue A, Koehler KM, Wayne SJ, Chiulli SJ, Haaland KY, Garry PJ. Nutritional status and cognitive functioning in a normally aging sample: a 6-y reassessment. The American journal of clinical nutrition. 1997;65(1):20-9.

17. Naurath HJ, Joosten E, Riezler R, Stabler SP, Allen RH, Lindenbaum J. Effects of vitamin B12, folate, and vitamin B6 supplements in elderly people with normal serum vitamin concentrations. Lancet. 1995;346(8967):85-9.

18. Deijen JB, van der Beek EJ, Orlebeke JF, van den Berg H. Vitamin B-6 supplementation in elderly men: effects on mood, memory, performance and mental effort. Psychopharmacology (Berl). 1992;109(4):489-96.
