# Supplementary material for: The preventive efficacy of vitamin B supplements on the cognitive decline of elderly adults: a systematic review and meta-analysis
Source: BMC Geriatr. 2021 Jun 16;21:367. doi: 10.1186/s12877-021-02253-3 (PMC8207668; doi:10.1186/s12877-021-02253-3)
Supplement: Supplementary file 5 — The Cochrane Risk of Bias Assessment. [file 12877_2021_2253_MOESM5_ESM.docx]

# Additional file 5: The Cochrane Risk of Bias Assessment

| Included studies | Random sequence generation | Allocation concealment | Blinding of participants and providers | Blinding of assessors | Incomplete outcome data | Selective reporting | Other bias |
| --- | --- | --- | --- | --- | --- | --- | --- |
| Fei Ma (2019) | low | unclear | low | high | low | unclear | unclear |
| Kwok Timothy (2019) | low | low | low | low | low | unclear | low |
| Fei Ma (2017) | low | unclear | high | high | low | unclear | unclear |
| Kwok Timothy (2017) | low | low | low | low | low | unclear | low |
| Cheng D (2016) | unclear | unclear | unclear | unclear | low | unclear | low |
| Dangour AD (2015) | low | low | low | low | low | unclear | low |
| van der Zwaluw NL (2014) | low | unclear | low | low | low | low | low |
| Hankey GJ (2013) | low | low | low | low | low | low | low |
| Walker JG (2012) | low | low | low | unclear | low | unclear | low |
| de Jager CA (2012) | unclear | low | low | low | low | low | low |
| Ford AH (2010) | low | low | low | low | low | low | low |
| Van Uffelen JG (2008) | low | low | low | low | low | low | low |
| Durga J (2007) | unclear | low | low | low | low | low | low |
| McMahon JA (2006) | low | low | low | unclear | low | unclear | low |
| Eussen SJ (2006) | low | low | low | unclear | low | unclear | low |
| Stott DJ (2005) | unclear | low | low | low | low | unclear | low |
| Lewerin C (2005) | unclear | low | low | unclear | low | unclear | low |
| Hvas AM (2004) | low | low | low | low | low | unclear | low |
| Garcia A (2004) | unclear | low | unclear | unclear | low | unclear | low |
| Janet Bryan (2002) | unclear | low | unclear | unclear | low | unclear | low |
| Fioravanti M (1997) | unclear | unclear | low | unclear | low | unclear | low |

*low risk of bias; unclear risk of bias; high risk of bias
